# Improved high sensitivity screen for Huntington disease using a one-step triplet-primed PCR and melting curve assay

**DOI:** 10.1371/journal.pone.0180984

**Published:** 2017-07-10

**Authors:** Mingjue Zhao, Felicia S. H. Cheah, Min Chen, Caroline G. Lee, Hai-Yang Law, Samuel S. Chong

**Affiliations:** 1 Department of Pediatrics, Yong Loo Lin School of Medicine, National University of Singapore, Singapore, Singapore; 2 Khoo Teck Puat – National University Children’s Medical Institute, National University Health System, Singapore, Singapore; 3 Department of Biochemistry, Yong Loo Lin School of Medicine, National University of Singapore, Singapore, Singapore; 4 Division of Medical Sciences, National Cancer Center, Singapore, Singapore; 5 Duke-NUS Graduate Medical School, Singapore, Singapore; 6 Department of Pediatric Medicine, KK Women’s and Children’s Hospital, Singapore, Singapore; 7 Department of Laboratory Medicine, National University Hospital, Singapore, Singapore; University of Minnesota Duluth, UNITED STATES

## Abstract

Molecular diagnosis of Huntington disease (HD) is currently performed by fluorescent repeat-flanking or triplet-primed PCR (TP-PCR) with capillary electrophoresis (CE). However, CE requires multiple post-PCR steps and may result in high cost in high-throughput settings. We previously described a cost-effective single-step molecular screening strategy employing the use of melting curve analysis (MCA). However, because it relies on repeat-flanking PCR, its efficiency in detecting expansion mutations decreases with increasing size of the repeat, which could lead to false-negative results. To address this pitfall, we have developed an improved screening assay coupling TP-PCR, which has been shown in CE-based assays to detect all expanded alleles regardless of size, with MCA in a rapid one-step assay. A companion protocol for rapid size confirmation of expansion-positive samples is also described. The assay was optimized on 30 genotype-known DNAs, and two plasmids p*HTT*(CAG)_26_ and p*HTT*(CAG)_33_ were used to establish the threshold temperatures (TTs) distinguishing normal from expansion-positive samples. In contrast to repeat-flanking PCR MCA, TP-PCR MCA displayed much higher sensitivity for detecting large expansions. All 30 DNAs generated distinct melt peak T_m_s which correlated well with each sample’s larger allele. Normal samples were clearly distinguished from affected samples. The companion sizing protocol accurately sized even the largest expanded allele of ~180 CAGs. Blinded analysis of 69 clinical samples enriched for HD demonstrated 100% assay sensitivity and specificity in sample segregation. The assay targets the *HTT* CAG repeat specifically, tolerates a wide range of input DNA, and works well using DNA from saliva and buccal swab in addition to blood. Therefore, rapid, accurate, reliable, and high-throughput detection/exclusion of HD can be achieved using this one-step screening assay, at less than half the cost of fluorescent PCR with CE.

## Introduction

Huntington Disease (HD; OMIM 143100) is an autosomal dominantly inherited progressive neurodegenerative disorder characterized by involuntary movement abnormality, cognitive loss and psychiatric manifestations and affects 5~10 persons per 100,000 in descents of western European [1]. The disease causing mutation is a CAG trinucleotide repeat expansion in exon 1 of the *Huntingtin* (*HTT*) gene located on chromosome 4p16.3 [2, 3]. *HTT* alleles are classified based on the number of CAG repeats: normal (≤ 26 CAGs), intermediate (27–35 CAGs), HD-causing with reduced-penetrance (36–39 CAGs), and HD-causing with full-penetrance (≥ 40 CAGs). Intermediate and expanded alleles have the tendency to increase in size during vertical transmissions [4, 5], with instability more pronounced in paternal transmission [4]. The age of on-set is inversely correlated with the number of CAG repeats. The clinical symptoms of HD progress gradually, usually with cognitive and psychiatric manifestations appearing first followed by movement abnormalities, and finally death [6, 7].

Predictive and diagnostic testing of HD require accurate sizing of the CAG repeat. PCR-based assays for sizing the *HTT* CAG repeat typically involve amplification using primers flanking the CAG repeat region, followed by capillary electrophoresis (CE) [8–10]. Whenever only a single peak is detected, additional tests such as PCR amplification of the adjacent CCG region and Southern blot are usually performed to exclude PCR amplification failure of large expanded alleles [11, 12]. The negative correlation between repeat length and amplification efficiency represents a significant deficiency of repeat-flanking PCR. Flanking sequence polymorphisms may also cause allele-specific PCR failure and lead to misdiagnosis [13–16]. In marked contrast, triplet primed PCR (TP-PCR), a strategy that pairs a flanking primer with one that anneals randomly within the repeat to generate different-sized amplicons, produces robust amplification and reliable detection of all expanded alleles regardless of size. This is because TP-PCR products of expanded alleles generate a characteristic CE pattern that can be easily distinguished from the pattern from non-expanded alleles [17], which eliminates the need to perform labour-intensive Southern blot. The TP-PCR strategy has been used to successfully detect an expanded allele of >200 CAG repeats [18], and to detect and size an expanded allele of ~180 CAG repeats [19]. The American College of Medical Genetics and Genomics committee has also indicated that TP-PCR is the preferred method for genetic testing of HD [20].

Between 1% and 7% of patients with HD-like features do not actually carry an expansion of the *HTT* CAG repeat, but may in fact be affected by other HD-like syndromes such as HD-Like (HDL) 1, 2, 3, the spinocerebellar ataxias (SCAs), Friedreich ataxia (FRDA), and dentatorubral-pallidoluysian atrophy (DRPLA), and most commonly amyotropic lateral sclerosis (ALS or Lou-Gehrig’s disease) or frontotemporal lobar degeneration (FTLD) caused by expansions in the *C9orf72* hexanucleotide repeat [20–26]. In some countries such as South Africa, HDL2 accounts for 24–50% of HD phenocopies [27]. Thus, having accurate methods to rapidly screen and differentiate between HD and HD-like diseases will save on diagnostic time and cost. Furthermore, although there is currently no cure for HD, a positive diagnosis is important for emotional relief and may help people come to practical decisions about careers, life and family planning. Given the availability of Tetrabenazine, the first FDA-approved drug for the treatment of chorea in HD [28], and clinical trials for HD treatment looming on the horizon [29–31], it would be useful to have a simple and cost-effective diagnostic strategy for asymptomatic individuals from at-risk families who request for testing to determine their genetic status. We previously described a cost-effective and rapid strategy to screen for HD expansion mutations based on melting curve analysis (MCA) of amplicons generated by repeat-flanking PCR [32]. However, PCR amplification across the repeat preferentially amplifies the smaller normal alleles at the expense of the larger expansions, which could potentially lead to a false negative result if an affected sample’s expansion is very large. We now describe an improved yet equally simple one-step screening assay involving MCA of TP-PCR products that, will detect all samples with an expansion regardless of the size of the expanded allele. Screen-positive samples can be rapidly confirmed by CE of the post-MCA TP-PCR product using a quick extension labelling step, thus avoiding the need to perform a separate fluorescent TP-PCR reaction on a separate aliquot of sample DNA.

## Materials and methods

### DNA samples

Genomic DNA was initially extracted from 12 HD-affected lymphoblastoid cell lines and 3 HD-unaffected cell lines purchased from Coriell Cell Repositories (CCR, Coriell Institute for Medical Research, USA) and used for assay optimization. An additional 15 DNAs including 14 HD reference DNAs with verified *HTT* genotypes [33] were also purchased from CCR and used for further validation. Sixty-nine archival DNA samples from KK Women’s and Children’s Hospital were included in a blinded evaluation of the screening assay’s accuracy. This study was reviewed and approved by the National University of Singapore Institutional Review Board (Ref: 07-123E) and the SingHealth Centralised Institutional Review Board (Ref: 2013/073/A).

### PCR and MCA conditions

Comparisons between repeat-flanking PCR MCA [32] and TP-PCR MCA were performed on the StepOnePlus^™^ Real-Time PCR System (Thermofisher Scientific, Massachusetts, USA). The sequences of the TP-PCR primers have been described elsewhere [19]. Other TP-PCR MCA assays were performed on a LightCycler^®^ 480 Real-Time PCR System (Roche Diagnostics, Germany). Each 25 μl PCR reaction mixture contained 10 ng genomic DNA, 1.5 x Q-Solution (Qiagen, Germany), 1 x PCR buffer containing 1.5 mM of MgCl_2_ (Qiagen), 0.2 mM deoxyribonucleic triphosphates (Roche Applied Science, Germany), 2 units of HotStar *Taq* DNA polymerase (Qiagen), 0.1 x SYBR^®^ Green I nucleic acid dye (Roche Applied Science), 0.5 μM of HD-F and TAIL primers, and 0.05 μM of TPP primer. PCR cycling conditions consisted of an initial polymerase activation step at 95°C for 15 min followed by 30 cycles of 98°C for 45 s, 63°C for 1min, and 72°C for 5 min. A melting curve program was automatically initiated immediately upon the completion of PCR, consisting of denaturation at 95°C for 1 min, a temperature-hold step at 60°C for 1 min, and a temperature ramping step from 60°C to 95°C at a rate of 0.8% in continuous mode (StepOnePlus^™^) or 0.01°C/sec with an acquisition of 50 fluorescence-intensity readings per degree Celsius change (Lightcycler^®^ 480). The data was analyzed using the respective instrument softwares.

### TP-PCR MCA performance parameters

Two pre-mutation (GM06892 and GM06907) and two full-mutation (GM06852 and GM07537) fragile X samples, as well as two myotonic dystrophy type 1 affected samples (GM05164 and GM06075) were used to test assay specificity. For testing the effect of input DNA amount, TP-PCR MCA reactions were identical except that 100 pg, 1 ng, 5 ng, 10 ng, 50 ng, 100 ng, 500 ng or 1 μg DNA was used. DNAs extracted from saliva and buccal swab were used to test the effect of different DNA sources. In addition, the effects of two common precipitants used in DNA extraction, glyocogen and sodium acetate, were tested. The TP-PCR MCA reactions were identical except that 0 μg, 5 μg, 10 μg and 20 μg glycogen, and 0 mM, 1 mM, 10 mM, 50 mM and 100 mM sodium acetate were added, respectively. All reactions were performed in triplicate.

### Allele sizing by capillary electrophoresis

Post-MCA allele sizing was accomplished using a short-cycle labelled-primer extension reaction. Each 20 μl reaction contained 2 μl of TP-PCR product, 1.5 x Q-Solution (Qiagen), 1 x PCR buffer containing 1.5 mM of MgCl_2_ (Qiagen), 0.25 mM dNTPs (Roche Applied Science), 1.25 units of HotStar *Taq* DNA polymerase (Qiagen, Germany), and 0.4 μM 6-carboxyfluorescein labelled HD-F primer. Cycling conditions were identical to those for TP-PCR MCA step except that only 5 cycles were used. A 2 μl aliquot of labelled extension product was mixed with 9 μl of Hi-Di^™^ formamide (Applied Biosystems, California, USA) and 0.5 μl of GeneScan^™^ 500 ROX^™^ dye size standard (Applied Biosystems). The mixture was denatured at 95°C for 5 min, cooled to 4°C, and resolved in a 3130xl Genetic Analyzer (Applied Biosystems) using a 36 cm capillary filled with POP4^™^ polymer. Samples were electrokinetically injected at 1 kV for 5 sec and electrophoresed for 2400 sec. GeneScan analysis was performed with GeneMapper 4.0 software (Applied Biosystems).

## Results

### Comparison between repeat-flanking PCR MCA and TP-PCR MCA

To compare the performance of the TP-PCR MCA assay with the published repeat-flanking PCR MCA assay, one normal and three HD-affected samples were analyzed in parallel using the previously published repeat-flanking PCR MCA method [32] and the new TP-PCR MCA method. Control plasmids p*HTT*(CAG)_26_ and p*HTT*(CAG)_33_ were used to generate threshold temperatures (TTs) for effective segregation of normal, intermediate, and expanded alleles.

**Fig 1:**
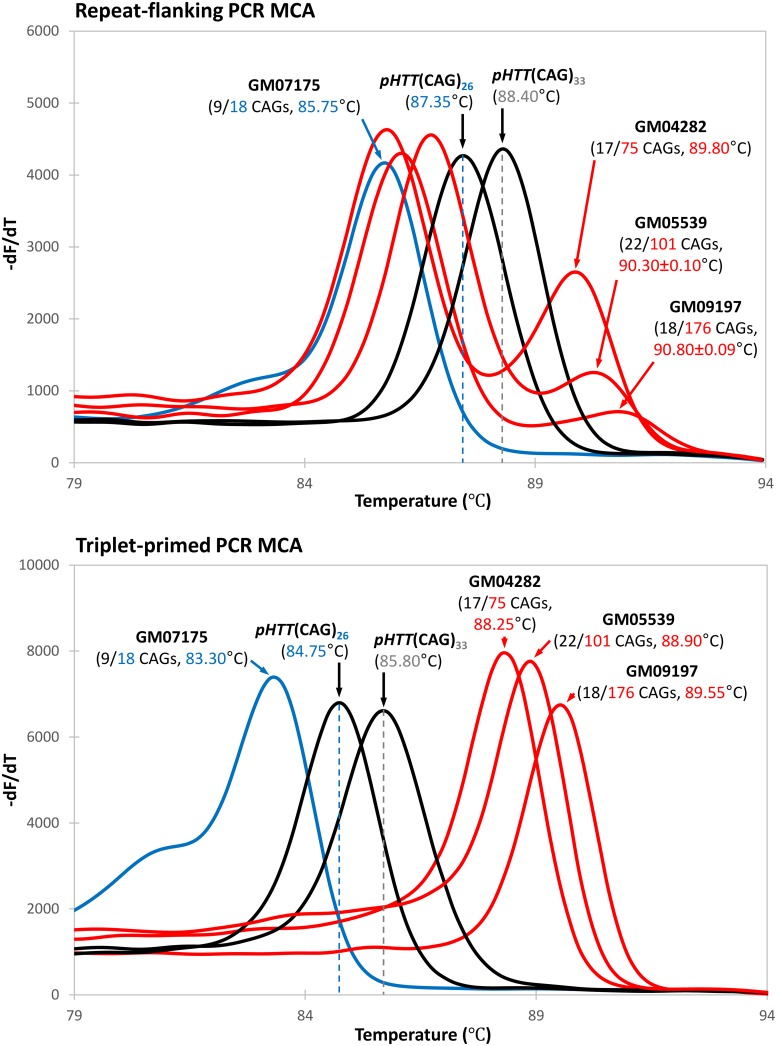
Comparison of repeat-flanking PCR and TP-PCR melt peaks. Melt peaks of normal and HD-affected samples are plotted in blue and red lines, respectively, while the p*HTT*(CAG)_26_ and p*HTT*(CAG)_33_ melt peaks are in black lines. Samples were assayed in triplicate. **Top**, repeat-flanking PCR MCA of the normal sample produces a single melt peak with a T_m_ in the normal range, whereas the HD-affected samples produce a dominant melt peak with T_m_ in the normal range and second melt peak with T_m_ in the expanded range. Melt peak height of the expanded allele decreases with increasing repeat length, risking an absent expanded allele peak if expansion is very large, with only the normal allele peak present. **Bottom**, TP-PCR MCA produces a single distinct melt peak in every sample regardless of disease status or length of repeat. The melt peak T_m_ relative to the threshold temperatures of p*HTT*(CAG)_26_ and p*HTT*(CAG)_33_ effectively determine normal or HD-affected status of each sample.

Repeat-flanking PCR MCA produced 1–2 melt peaks depending on the CAG repeat sizes of the two alleles and the size difference between the two alleles (Fig 1, top panel). The normal sample GM07175 (with 9 and 18 CAGs) produced a single melt peak with a T_m_ of 85.75°C, which is lower than the 87.35°C TT of the 26 CAG repeat control plasmid p*HTT*(CAG)_26_. In contrast, the HD-affected samples GM04282 (17 and 75 CAGs), GM05539 (22 and 101 CAGs) and GM09197 (18 and 176 CAGs) produced two melt peaks, with the lower T_m_ melt peak representing the normal allele and the higher T_m_ melt peak representing the expanded allele. The expanded allele melt peaks had T_m_s that were higher than the 88.40°C TT of the 33 CAG repeat control plasmid p*HTT*(CAG)_33_.

**Fig 2:**
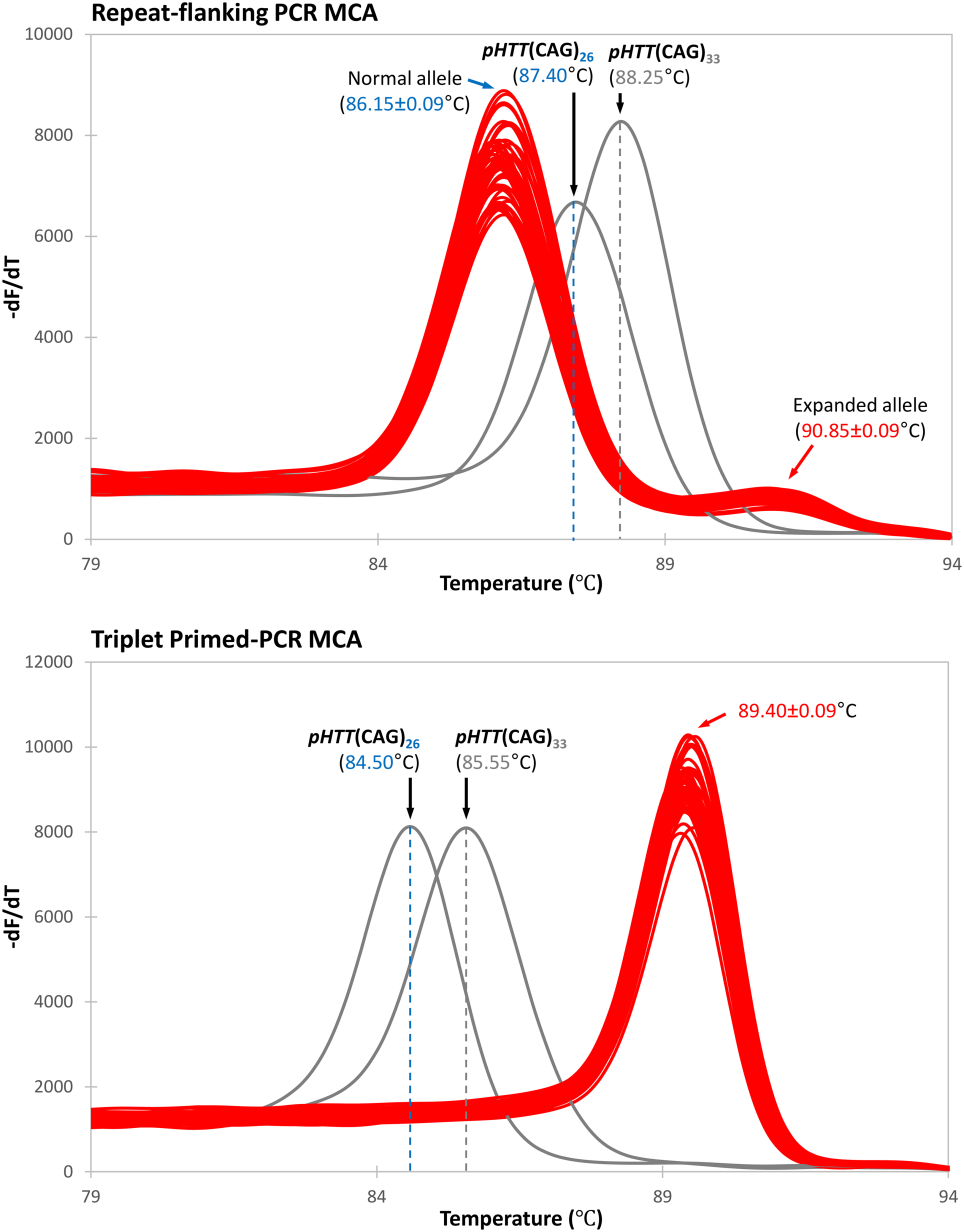
Reproducibility of repeat-flanking PCR and TP-PCR melt peaks. Sample GM09197, which carries an expanded allele of ~180 CAG repeats, was assayed in parallel by repeat-flanking PCR MCA and TP-PCR MCA. Forty-eight replicates of each assay were performed. Melt peaks of replicates are plotted in red, while the melt peaks of the control plasmids p*HTT*(CAG)_26_ and p*HTT*(CAG)_33_ are in black. **Top**, using repeat-flanking PCR MCA, the expanded allele melt peak is much weaker than the normal allele and is almost flat, making result interpretation ambiguous. **Bottom**, using TP-PCR MCA, a highly reproducible and distinct single melt peak is observed, with a T_m_ clearly in the HD-affected range.

However, it was observed that the melt peaks of the expanded alleles were of much lower peak height compared to their corresponding normal alleles. Their peak heights were also observed to be inversely proportional to the repeat size of the expanded allele. Notably, the melt peak for the 176 CAG repeat expanded allele of GM09197 was almost flat (Fig 1 top panel), and this observation was highly reproducible (Fig 2, top panel). These observations highlight the major deficiency of repeat-flanking PCR, which is the decreasing amplification efficiency with increasing repeat size. There is thus a potential but real risk of a false-negative screen result when an HD-affected sample carries an expanded CAG repeat large enough to suppress amplicon yield to the point that an expanded allele melt peak is not detected. Therefore, the larger an expanded allele, the lower its detection sensitivity is when using repeat-flanking PCR MCA.

Unlike repeat-flanking PCR MCA, TP-PCR MCA produces only one melt peak regardless of whether a sample is normal or HD-affected, and effectively detects the presence of an expanded allele through the observation of a right-shifted melt peak (Fig 1, bottom panel). The 83.30°C melt peak T_m_ of the normal sample GM07175 (9/18 CAGs) was lower than the 84.75°C TT of control plasmid p*HTT*(CAG)_26_. In marked contrast, the melt peak T_m_s of HD-affected samples GM04282 (17/75 CAGs), GM05539 (22/101 CAGs) and GM09197 (18/176 CAGs) were greater than the 85.80°C TT of control plasmid p*HTT*(CAG)_33_.

Most importantly, strong and tall peak heights were observed for all three HD-affected samples (Fig 1 bottom panel), and the melt peaks were highly reproducible (Fig 2, bottom panel), allowing consistently unequivocal classification of their HD affected status regardless of how large the expanded allele was. These data demonstrate that TP-PCR MCA is more robust and reliable compared to repeat-flanking PCR MCA when used as a tool for rapid high throughput screening of Huntington disease.

### Correlation between TP-PCR melt peak T_m_ and larger allele size

**Fig 3:**
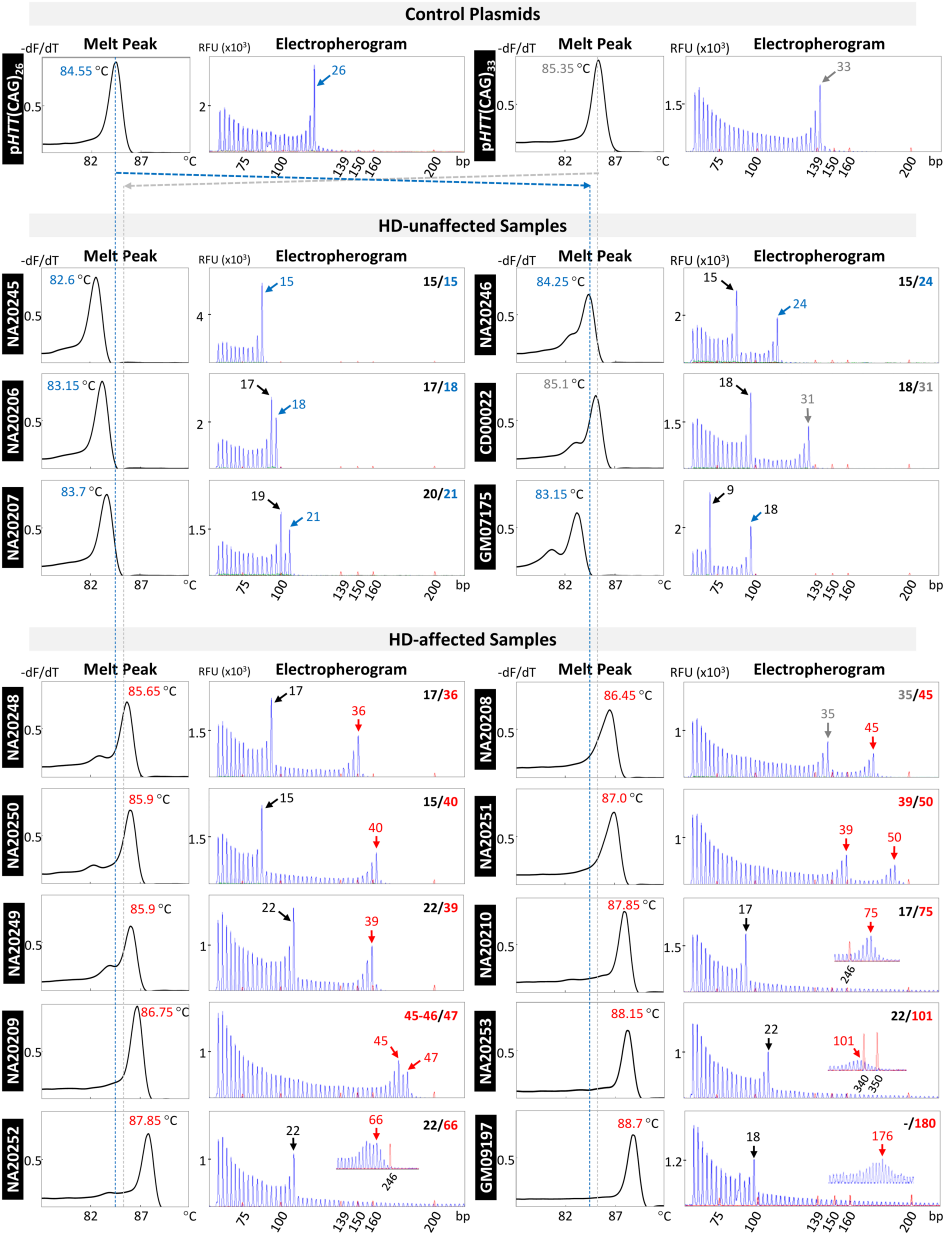
TP-PCR melt peaks and capillary electropherograms of genotype-known CCR (Coriell Cell Repositories) samples. For all samples, melt peak temperature correlated well with repeat length of the larger allele. Verified or CCR-provided genotypes are indicated at the upper right corner of each electropherogram. Allele sizes determined from TP-PCR capillary electrophoresis are indicated by arrows. Insets show magnified view of expanded alleles. For all samples, the allele sizes and genotypes determined using TP-PCR assay were concordant with the verified allele sizes.

**Fig 4:**
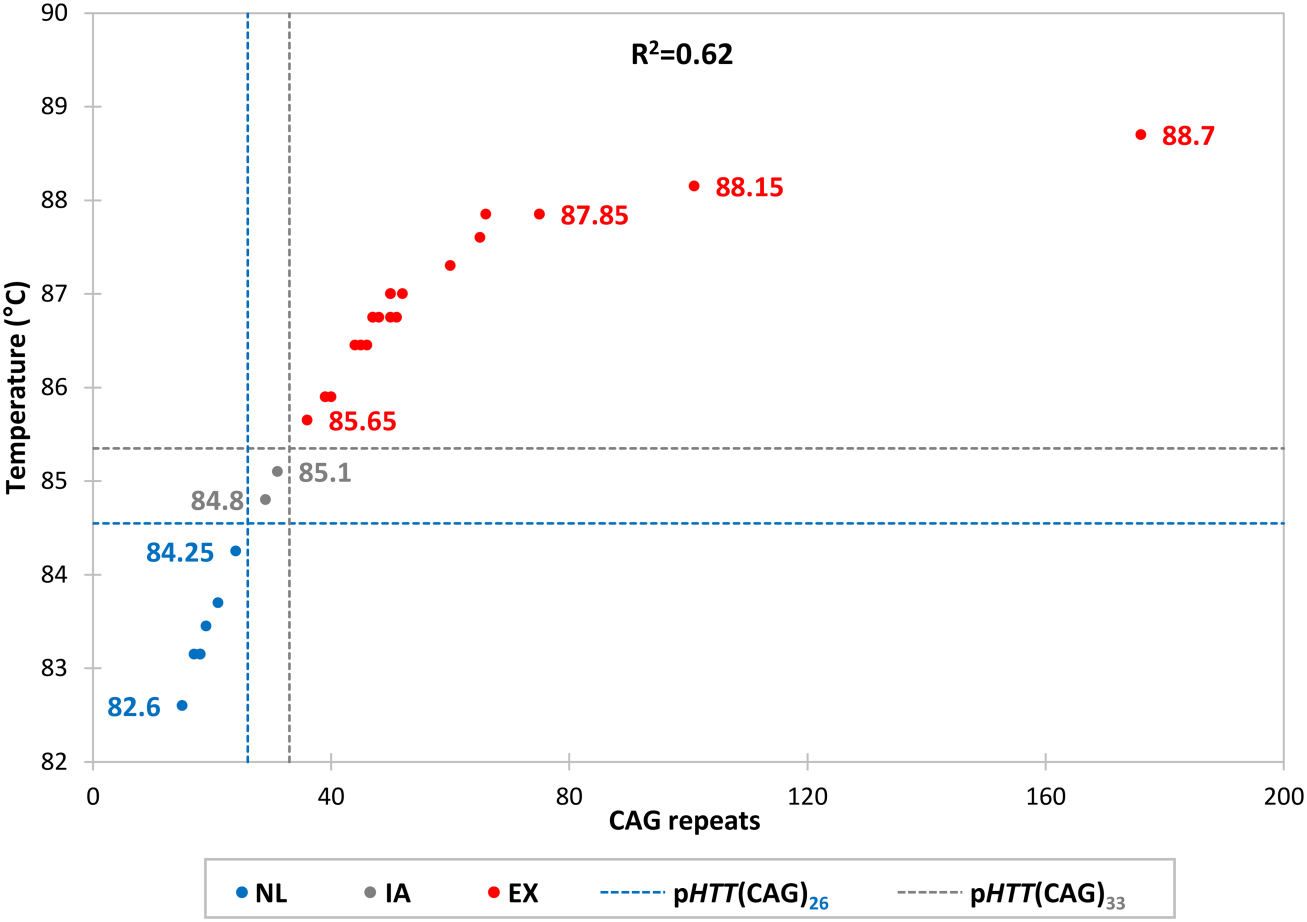
Correlation of TP-PCR melt peak temperature with CAG repeat size of the larger allele. NL, sample carrying only normal alleles; IA, sample carrying an intermediate allele; EX, sample carrying an expanded allele. A good correlation was observed between the TP-PCR melt peak T_m_ and the CAG repeat size of the larger allele among the samples, allowing unambiguous discrimination between normal and HD-affected samples.

We performed TP-PCR MCA, followed by labelled-primer extension and capillary electrophoresis of the TP-PCR amplicons, using genomic DNA of 30 cell lines obtained from the Coriell Cell Repositories (CCR). Of these, 21 were from HD-affected individuals and 9 were from HD-unaffected individuals. Representative TP-PCR melt peak profiles of 10 HD-affected and 6 unaffected CCR samples are shown in Fig 3. Also shown beside each melt peak are the corresponding GeneScan electropherograms that were generated by short-cycle labelled-primer extension of the TP-PCR amplicons followed by capillary electrophoresis. The genotypes determined using the TP-PCR MCA extension labelling were completely concordant with the verified sizes of the reference panel samples [33] (Table 1), validating the accuracy of the TP-PCR assay. Samples with alleles of ≤31 CAGs produced melt peak temperatures of ≤85.1°C whereas samples with an expanded allele ≥36 CAGs generated melt peak temperatures of ≥85.65°C, showing good correlation between size of the larger allele in the sample and the TP-PCR melt peak temperature, with sufficient T_m_ discrimination between HD-affected and unaffected samples (Fig 4).

**Table 1:** Correspondence of the CAG repeat size of the larger allele obtained from this TP-PCR CE analysis with previously verified allele sizes.

Sample ID	CCR (CAG)_n_	GeT-RM (CAG)_n_ ✝				TP-PCR (This study)
		Mean	Modal	Sequencing		CE (CAG)_n_
GM07426*	N.d.	N.d.				17
GM07175*						18
GM10798*						19
GM04479**	42					44
GM04866**	44					46
GM04477**	45					47
GM05626**	47					48
GM04856**	50					50
GM06528**	51					50
GM05542**	52					51
GM06581**	53					52
GM03620	N.d.					60
GM04738**	65					65
GM04282**	69					75
NA20245	15†	15	15		15	15
NA20206	18†	18	18		18	18
NA20207	21†	21	21		21	21
NA20246	24†	24	24		24	24
NA20247	29†	29	29		29	29
CD00022	31†	N.d.				31
NA20248	36†	36	36		36	36
NA20249	39†	39	39		39	39
NA20250	40†	40	40		40	40
NA20208	45†	45	45		45	45
NA20209	47†	47	47		46	47
NA20251	50†	50	50		50	50
NA20252	66†	66	65–66		65	66
NA20210	75†	74	74		75	75
NA20253	101†	99	100		101	101
GM09197	180†	N.d.				176

^✝^Sample IDs with an NA prefix have been verified by the GeT-RM (Genetic Testing Reference Materials coordination program [33] and designated as reference genotype samples. Mean allele size was calculated from the average of all reported allele sizes determined by participating diagnostic labs. Modal allele size was the most common reported allele size. Sequencing allele size was obtained from unidirectional sequencing across the repeat.

Except those sample IDs with a * symbol, all other samples in the Table belong to the CCR (Coriell Cell Repositories) HD disease category.

** Allele sizes were obtained from previous internal testing using repeat-flanking PCR.

^†^ Allele sizes were obtained from CCR.

N.d. Not determined

### Classification of disease status using melt peak temperature

**Fig 5:**
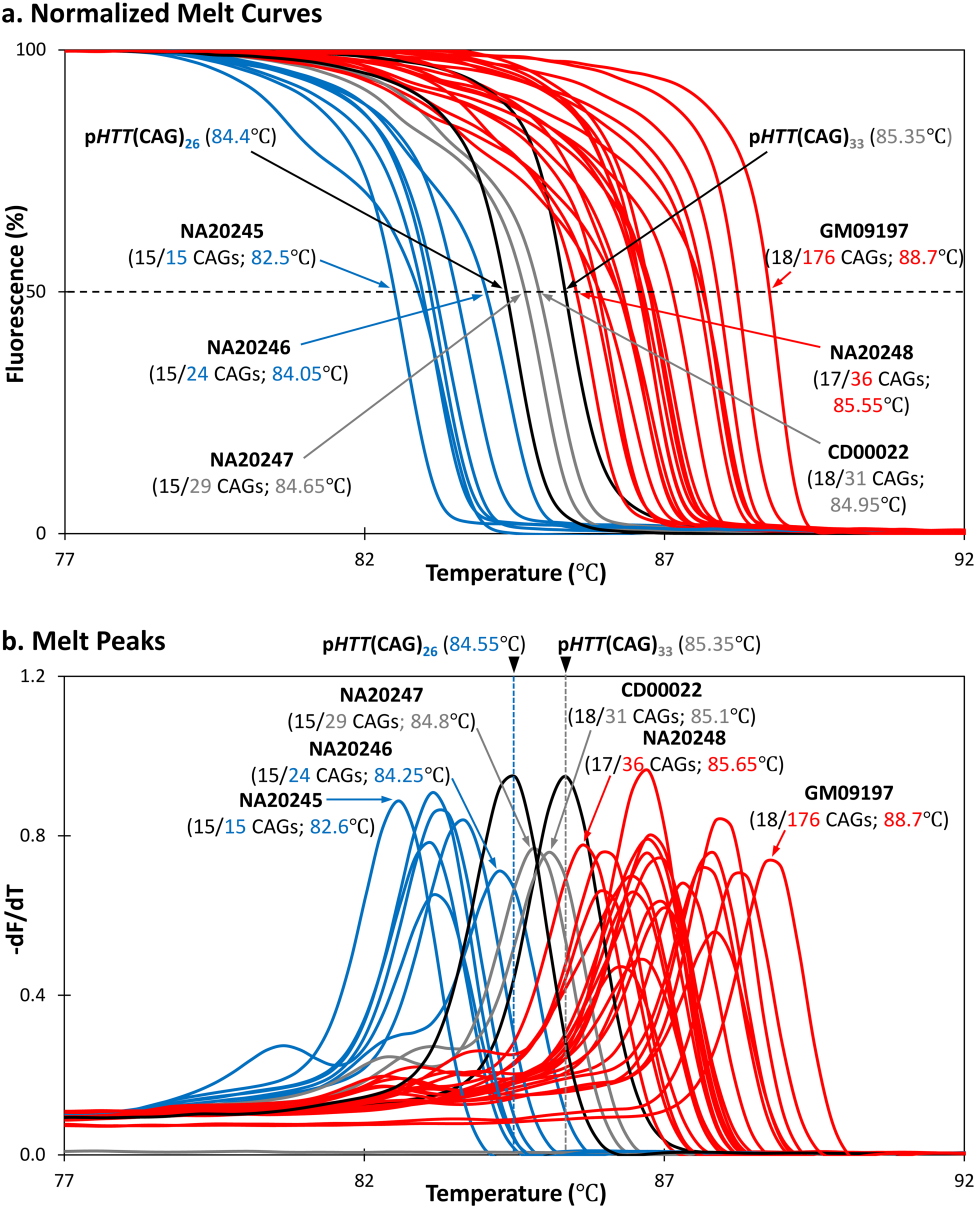
Normalized melt curves and melt peaks of 30 genotype-known CCR samples. Samples harboring normal-only, intermediate and expanded alleles are plotted in blue, grey and red lines, respectively. Based on the T_m_s of the samples relative to the threshold temperatures generated by p*HTT*(CAG)_26_ and p*HTT*(CAG)_33_, all CCR samples were correctly classified.

Fig 5 displays the normalized initial melt curves (Fig 5a) and derivative melt peaks (Fig 5b) of all 30 CCR samples relative to the melt peaks of p*HTT*(CAG)_26_ and p*HTT*(CAG)_33_, which were included in each plate/run to control for minor inter-run variations in T_m_ values. Using either melt curve or melt peak plot, all 30 samples were classified correctly with reference to the p*HTT*(CAG)_26_ and p*HTT*(CAG)_33_ TTs in both plots. The very large expanded allele in GM09197 (~180 CAG repeats) was also successfully sized by CE after extension labeling of the TP-PCR MCA product (Fig 3). In summary, TP-PCR MCA effectively detects the presence of an expanded allele in a DNA sample, through the observation of a right-shifted melt curve/peak with a T_m_ that is higher than the TT established by p*HTT*(CAG)_33_ within the same run.

### Blinded clinical sample validation

**Fig 6:**
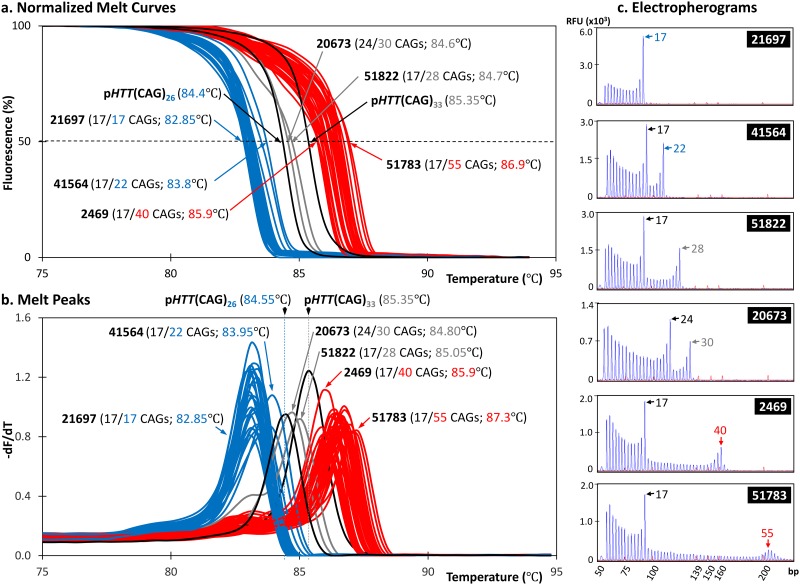
TP-PCR MCA profiles of 69 clinical samples enriched for Huntington disease. Samples harboring normal-only, intermediate and expanded alleles are plotted in blue, grey and red lines, respectively. The T_m_s and corresponding capillary electropherograms of two normal, two IA and two HD-affected samples are shown. Based on the T_m_s of the samples relative to the threshold temperatures generated by p*HTT*(CAG)_26_ and p*HTT*(CAG)_33_, all 69 clinical samples were correctly classified.

To evaluate the sensitivity and specificity of TP-PCR MCA in distinguishing normal from HD-affected samples, a blinded test was performed using 69 clinical samples that had previously been genotyped at the *HTT* CAG repeat locus, consisting of 29 normal and 40 HD-affected individuals. TP-PCR MCA results were confirmed by rapid short-cycle primer extension of an aliquot of TP-PCR product followed by capillary electrophoresis. Normalized melt curves and derivative melt peaks of all 69 samples, as well as the GeneScan electropherograms of representative normal and HD-affected samples are shown in Fig 6. All samples (40 HD-affected, 2 intermediate, and 27 normal) were correctly classified by TP-PCR MCA, and results were completely concordant with the GeneScan electropherogram-derived genotypes (S1 Table).

### Demarcation of TP-PCR MCA performance parameters

**Fig 7:**
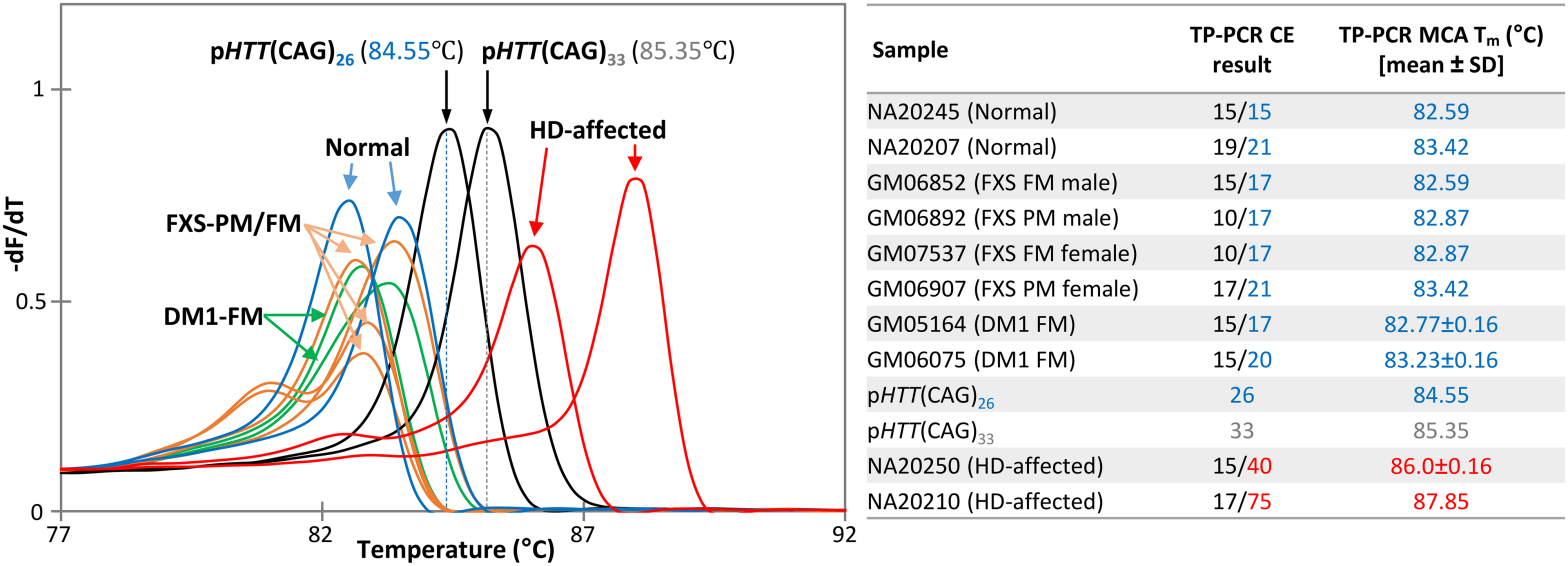
Analytic specificity of the TP-PCR MCA assay. Analytic specificity was assessed by performing the assay on samples carrying premutation or full mutation *FMR1* alleles (FXS-PM/FM) or full mutation *DMPK* alleles (DM1-FM), together with HD-normal and HD-affected samples as controls. The TP-PCR melt peak temperatures of the 4 samples carrying FXS premutations or full mutations, and the 2 samples carrying DM1 expansions, were observed to be lower than the TT of the control plasmid p*HTT*(CAG)_26_. The absence of any T_m_ higher than the TT of p*HTT*(CAG)_33_ in these samples indicates the absence of non-specific amplification at the FXS and DM1 repeat loci. These results indicate that the *HTT* TP-PCR MCA assay is specific for the Huntington disease locus.

To evaluate the locus specificity of the *HTT* TP-PCR MCA assay, 6 samples with expansion mutations at the *FMR1* CGG repeat locus responsible for fragile X syndrome (FXS), or the *DMPK* CTG repeat locus responsible for myotonic dystrophy type 1 (DM1), were tested. These samples carry either a premutation *FMR1* allele such as GM06892 (male with 93 CGG repeats) and GM06907 (female with 29/91 CGG repeats), a full mutation *FMR1* allele such as GM06852 (male with >200 repeats) and GM07537 (female with 29/>200 CGG repeats), or a full mutation *DMPK* allele such as GM06075 (12/56-70 CTG repeats) and GM05164 (21/377 CTG repeats). The TP-PCR melt peak temperatures of all 6 samples were observed to be lower than the TT of the control plasmid p*HTT* (CAG)_26_ (Fig 7), thus falling in the normal T_m_ range. This indicates that the *HTT* TP-PCR assay amplified only at the HD locus and not at the FXS or DM1 loci. This was confirmed by subsequent extension labelling and CE analysis of the TP-PCR products, which showed genotypes of 10/17, 17/21, 15/17 and 10/17 for the FXS samples and 15/20 and 15/17 for the DM1 samples. These results demonstrate the specificity of the TP-PCR MCA for the HD locus.

**Fig 8:**
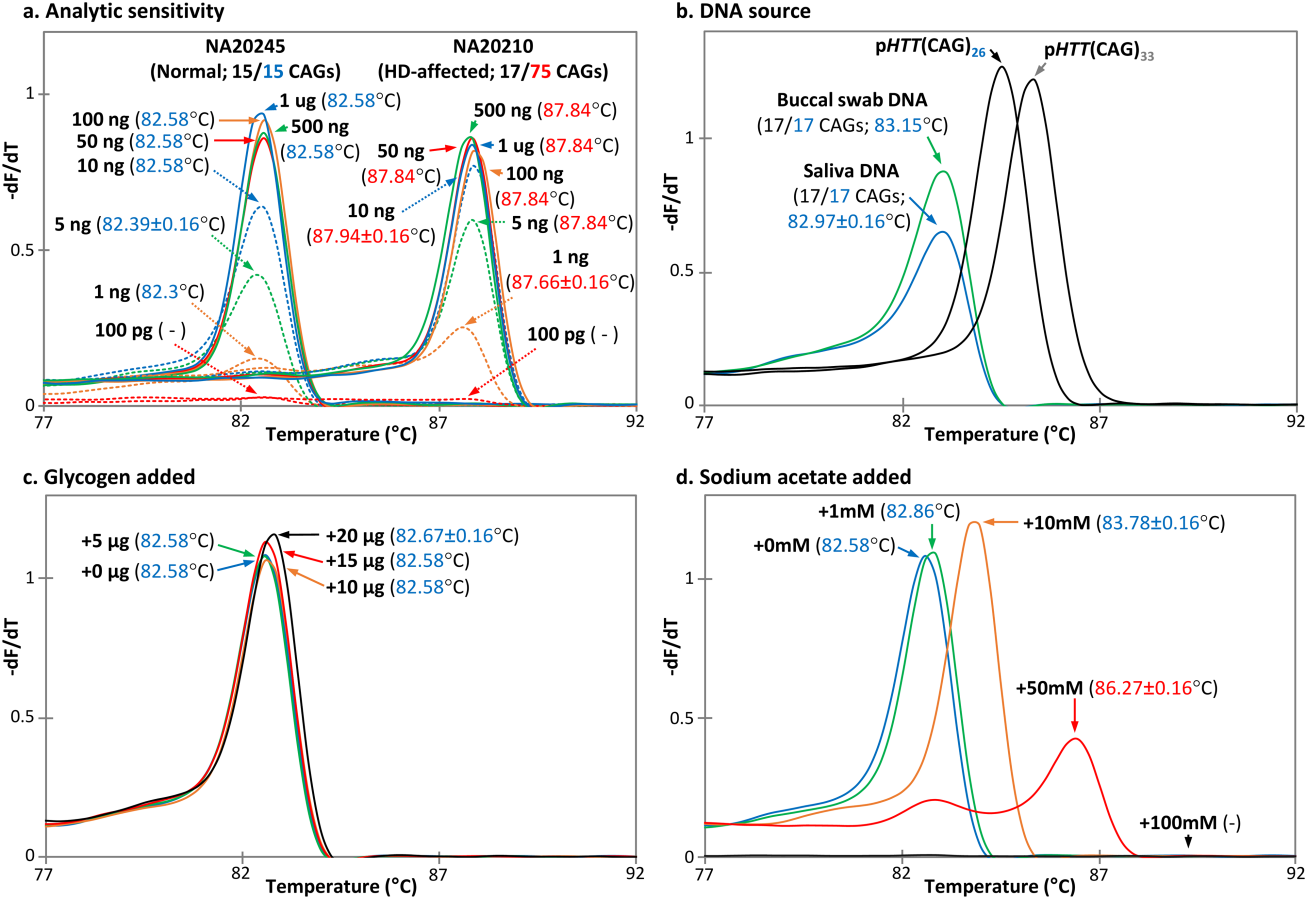
Performance characteristics of the TP-PCR MCA assay. Analytic sensitivity was determined over an input DNA range of 100 pg to 1 μg. Accurate sample classification was achieved using input DNA of 10 ng to 1 μg (**a**). The assay performed equally well using DNA extracted from blood, buccal swab, or saliva (**b**). The presence of glycogen of up to 20 μg did not adversely affect the assay (**c**), but increasing amounts of sodium acetate contamination produced progressively right-shifted melt peak temperatures, and the assay was inhibited at 100 mM concentration (**d**). All experiments were performed in triplicate.

To test the sensitivity of the TP-PCR MCA assay, input DNA amounts ranging from 1 ng to 1 μg were tested and they produced similar melt peak temperatures (Fig 8a), although input DNA amounts of 1–5 ng resulted in reduced amplification yield. A distinct melt peak was not observed at 100 pg of input DNA. DNAs extracted from other sources such as saliva and buccal swab were also tested, and they showed no observable adverse effect on assay performance (Fig 8b). Given that precipitants are frequently used during extraction of limiting amounts of DNA, we investigated whether presence of any carryover of precipitants in DNA solutions could affect the melt peak T_m_s. We performed TP-PCR MCA in the presence of two common DNA precipitants, glycogen and sodium acetate, at different concentrations. There was no observable effect of glycogen, at any of the tested concentrations, on either melt peak height or temperature (Fig 8c). In contrast, with increasing sodium acetate concentrations, melt peak T_m_s were observed to be progressively right-shifted, thus potentially altering a sample’s disease classification especially at higher salt concentrations (Fig 8d). In addition, higher salt concentrations also progressively inhibited the reaction.

## Discussion

Accurate detection of CAG repeat expansion within the *HTT* gene, particularly around the diagnostic transition range, is crucial for the diagnostic and predictive testing of HD [34] as well as for the differential diagnosis of HD and HD-like diseases [35]. Capillary electrophoresis (CE) of fluorescent TP-PCR products simplifies repeat sizing of all normal and most expanded alleles without reliance on sizing standards, by simple counting of the number of amplicon peaks that are generated in the electropherogram. Most importantly, this method will reliably detect the presence of an expanded allele regardless of the size of expansion. However, CE is relatively labor-intensive and expensive if used in the context of large scale screening, when compared to more homogeneous methods such as melt curve analysis (MCA). We previously demonstrated the utility of using MCA for molecular screening of HD [32]. However, because of its reliance on repeat-spanning PCR, a major pitfall of the assay was the poor amplification efficiency for larger alleles, which risked a failure to detect very large expanded alleles when present and a false negative misdiagnosis. In this study, we have coupled TP-PCR with MCA to develop an improved, inexpensive yet accurate one-step assay to rapidly rule in/out HD. Comparisons with the repeat-flanking PCR MCA assay showed that the TP-PCR MCA assay has a better detection sensitivity for expanded alleles, with accurate sizing of the expanded allele of up to at least ~180 CAG repeats directly from the TP-PCR MCA product, similar to what has been achieved with TP-PCR and CE directly from genomic DNA [19]. We also showed that samples carrying only normal alleles produced normalized melt curves and derivative melt peaks with reproducibly lower T_m_s compared to samples carrying an expanded allele. TP-PCR MCA is thus more reliable than repeat-flanking PCR MCA in screening for HD-affected samples, while being just as cost-effective and amenable to high throughput applications, thus obviating the need to rely on the relatively costly fluorescent GeneScan analysis for every test sample, and limiting CE analysis to repeat sizing confirmation of only the expansion-positive samples.

To simplify the classification of screen-positive and screen-negative samples, control plasmids p*HTT*(CAG)_26_ and p*HTT*(CAG)_33_ allele were included in each run/plate to establish the discriminating threshold temperatures (TTs). A 33-repeat allele was determined to be optimal for establishing the upper TT because under repeated testing, the sample carrying the smallest expanded allele of 36 repeats was correctly identified as screen-positive. The 26-repeat allele was selected to establish the lower TT due to its ability to consistently discriminate between and correctly classify the samples in our cohort carrying the largest normal allele of 24 repeats and the smallest intermediate allele of 28 repeats.

It is possible that, with the use of a 33-repeat plasmid, a sample carrying an intermediate allele of 33–35 CAGs could be erroneously classified as expansion-positive. We therefore surveyed the published literature on the size distribution of *HTT* CAG repeats in the European, Chinese, African, Indian, and North and South American populations (S1 Fig). The largest intermediate alleles of 33–35 repeats account for ~0.28% of all alleles (exact counts were unavailable in some reports, in which case approximations were derived from the graphical data). Hence, the TT established by the p*HTT*(CAG)_33_ control plasmid provides 100% screening sensitivity (0% false negative rate) in identifying all HD-affected samples (i.e. those carrying an expanded allele of ≥36 CAG repeats), while retaining a >99.7% screening specificity (<0.3% chance of identifying an intermediate allele carrier as being expansion-positive (i.e. HD-affected). Samples with a higher melting temperature than the p*HTT*(CAG)_33_ generated TT should be defined as possible expansion-positives and should be confirmed by CE analysis.

Furthermore, we showed that the TP-PCR MCA assay is highly specific for the *HTT* CAG repeat expansion, produces good melt peaks over an input genomic DNA range of 10 ng to 1 μg, and works equally well with DNA extracted from blood, saliva or buccal swab (Figs 7 and 8). However, the assay’s accuracy is compromised in the presence of sodium acetate, where increasing concentrations of sodium acetate were observed to cause progressive right-shifting of the melt peak and thus melt temperature. The increase in melt peak temperature could be explained by an increased difficulty in double strand DNA denaturation in the presence of higher salt concentrations, culminating in complete amplification failure at very high salt concentrations. To minimize the impact of sodium acetate contamination on assay accuracy, care should be exercised to ensure that all high-throughput screening samples are processed identically and with minimal salt carryover.

Given the ethical controversies and current genetic testing guidelines for HD, the TP-PCR MCA assay described here is not proposed to be used to screen at-risk individuals who are minors. Nonetheless, the assay can act as a rapid and cost-effective genetic test for HD, especially as a first-tier tool to differentiate HD-like diseases from HD. It could be especially beneficial for centralized neurogenetics referral laboratories that perform testing on large numbers of patient samples with HD and HD-like phenotypes where test costs are a concern. Assuming an expected expansion-positive frequency of one HD-affected sample for every 10 samples tested in a molecular diagnostic lab, 10 TP-PCR CE tests will cost ~USD32, while 10 TP-PCR MCA tests (~USD13) and one follow-up sizing confirmation (~USD2.8) will cost a total of ~USD16. Even when as many as 6 of 10 samples tested turn out to be expansion-positive (HD-affected) and are followed-up with sizing confirmation, it will still be more cost-effective (~USD30) than performing 10 TP-PCR CE runs.

As a melt temperature based assay, TP-PCR MCA is not able to determine exact allele sizes in a DNA sample. However, while current guidelines recommend reporting the CAG repeat size of both alleles, it is not an absolute necessity to provide the allele sizes for normal samples, and only the allele sizes of intermediate allele carriers and HD-affected samples are important and should be provided.

This cost-effective single-step, closed-tube and scalable strategy can be easily applied for rapid screening of other choreo-ataxic disorders caused by trinucleotide repeat expansions, in particular the spinocerebellar ataxias (SCAs), Friedreich ataxia (FRDA) and dentatorubralpallidoluysian atrophy (DRPLA).

## Supporting information

S1 Table Results of blinded TP-PCR MCA screen of clinical samples and subsequent size confirmation by labelled-primer extension and capillary electrophoresis. (PDF)

S1 Fig *HTT* CAG allele frequency in 6 populations: African, South American, North American, European, Chinese and Indian (references are listed). Although alleles of 33–35 CAGs may be classified as screen-positive when using p*HTT*(CAG)_33_ to establish the threshold temperature, such alleles represent only ~0.28% of total alleles in the population, and screen-positive samples require a second-tier CE-based sizing analysis in order to be confirmed as being HD-affected.
(PDF)
