# Supplementary material for: Improved high sensitivity screen for Huntington disease using a one-step triplet-primed PCR and melting curve assay
Source: PLoS One. 2017 Jul 10;12(7):e0180984. doi: 10.1371/journal.pone.0180984 (PMC5507316; doi:10.1371/journal.pone.0180984)
Supplement: S1 Table — (PDF) [file pone.0180984.s001.pdf]

Supplemental Table S1. **Results of blinded TP-PCR MCA screen of clinical samples and subsequent size confirmation by labeled-primer extension and capillary electrophoresis.**

Sample ID	TP-PCR MCA screen		$(CAG)_n$ Genotype
	T_m (℃)	Result*	
61721	86.45	Expansion-Positive	17 / 44
62914	86.73	Expansion-Positive	17 / 49
50841	86.46	Expansion-Positive	17 / 46
51783	87.29	Expansion-Positive	17 / 55
72060	86.17	Expansion-Positive	19 / 40
10030	86.73	Expansion-Positive	17 / 49
10060	86.18	Expansion-Positive	17 / 41
10061	86.18	Expansion-Positive	17 / 41
21715	86.73	Expansion-Positive	17 / 48
30296	86.46	Expansion-Positive	28 / 46
20034	86.73	Expansion-Positive	20 / 48
21184	86.18	Expansion-Positive	24 / 42
21195	86.46	Expansion-Positive	17 / 47
257	86.46	Expansion-Positive	17 / 43
1685	87.28	Expansion-Positive	15 / 55
990350	86.73	Expansion-Positive	17 / 48
10552	86.45	Expansion-Positive	17 / 43
11098	86.73	Expansion-Positive	18 / 50
11099	87.01	Expansion-Positive	18 / 56
11571	85.9	Expansion-Positive	18 / 40
980740	86.73	Expansion-Positive	17 / 48
981108	86.18	Expansion-Positive	16 / 42
981043	86.18	Expansion-Positive	17 / 41
990314	86.45	Expansion-Positive	17 / 43
990348	86.73	Expansion-Positive	17 / 49
5385	86.45	Expansion-Positive	17 / 43
5957	86.46	Expansion-Positive	17 / 43
6027	86.74	Expansion-Positive	17 / 46
6236	86.73	Expansion-Positive	17 / 46
6644	87.01	Expansion-Positive	17 / 52
2228	86.46	Expansion-Positive	17 / 45
2469	85.9	Expansion-Positive	17 / 40
3926	86.45	Expansion-Positive	17 / 43
4354	86.46	Expansion-Positive	19 / 43

4577	86.73	Expansion-Positive	17 / 49
348	86.73	Expansion-Positive	17 / 49
393A	86.17	Expansion-Positive	17 / 41
888	86.46	Expansion-Positive	19 / 44
1056	86.17	Expansion-Positive	17 / 42
1359	86.73	Expansion-Positive	19 / 48
70244	83.13	Expansion-Negative	17 / 18
70457	83.41	Expansion-Negative	17 / 21
71017	83.4	Expansion-Negative	17 / 19
71764	83.12	Expansion-Negative	17 / 18
72341	83.12	Expansion-Negative	17 / 19
60390	83.13	Expansion-Negative	17 / 17
62605	83.12	Expansion-Negative	17 / 17
63211	83.41	Expansion-Negative	18 / 18
63212	83.13	Expansion-Negative	17 / 18
63234	83.12	Expansion-Negative	17 / 18
50468	83.41	Expansion-Negative	17 / 19
51822	85.07	Intermediate	17 / 28
51847	83.4	Expansion-Negative	17 / 20
52141	83.13	Expansion-Negative	17 / 18
52503	83.13	Expansion-Negative	17 / 17
42039	83.13	Expansion-Negative	17 / 17
50016	83.12	Expansion-Negative	17 / 18
50017	83.13	Expansion-Negative	17 / 19
50149	83.12	Expansion-Negative	17 / 18
50289	83.4	Expansion-Negative	17 / 19
21697	82.86	Expansion-Negative	17 / 17
30407	83.13	Expansion-Negative	18 / 18
30408	83.12	Expansion-Negative	17 / 18
41564	83.95	Expansion-Negative	17 / 22
20412	83.13	Expansion-Negative	17 / 18
20673	84.79	Intermediate	24 / 30
21073	83.69	Expansion-Negative	17 / 22
21400	83.13	Expansion-Negative	18 / 18
21462	83.12	Expansion-Negative	17 / 17

* Threshold temperatures of 84.55 °C and 85.35 °C were established from plasmids p*HTT*$(CAG)_{26}$ and p*HTT*$(CAG)_{33}$
