# Supplementary material for: Improved high sensitivity screen for Huntington disease using a one-step triplet-primed PCR and melting curve assay
Source: PLoS One. 2017 Jul 10;12(7):e0180984. doi: 10.1371/journal.pone.0180984 (PMC5507316; doi:10.1371/journal.pone.0180984)
Supplement: S1 Fig — Although alleles of 33–35 CAGs may be classified as screen-positive when using pHTT(CAG)33 to establish the threshold temperature, such alleles represent only ~0.28% of total alleles in the population, and screen-positive samples require a second-tier CE-based sizing analysis in order to be confirmed as being HD-affected. (PDF) [file pone.0180984.s002.pdf]

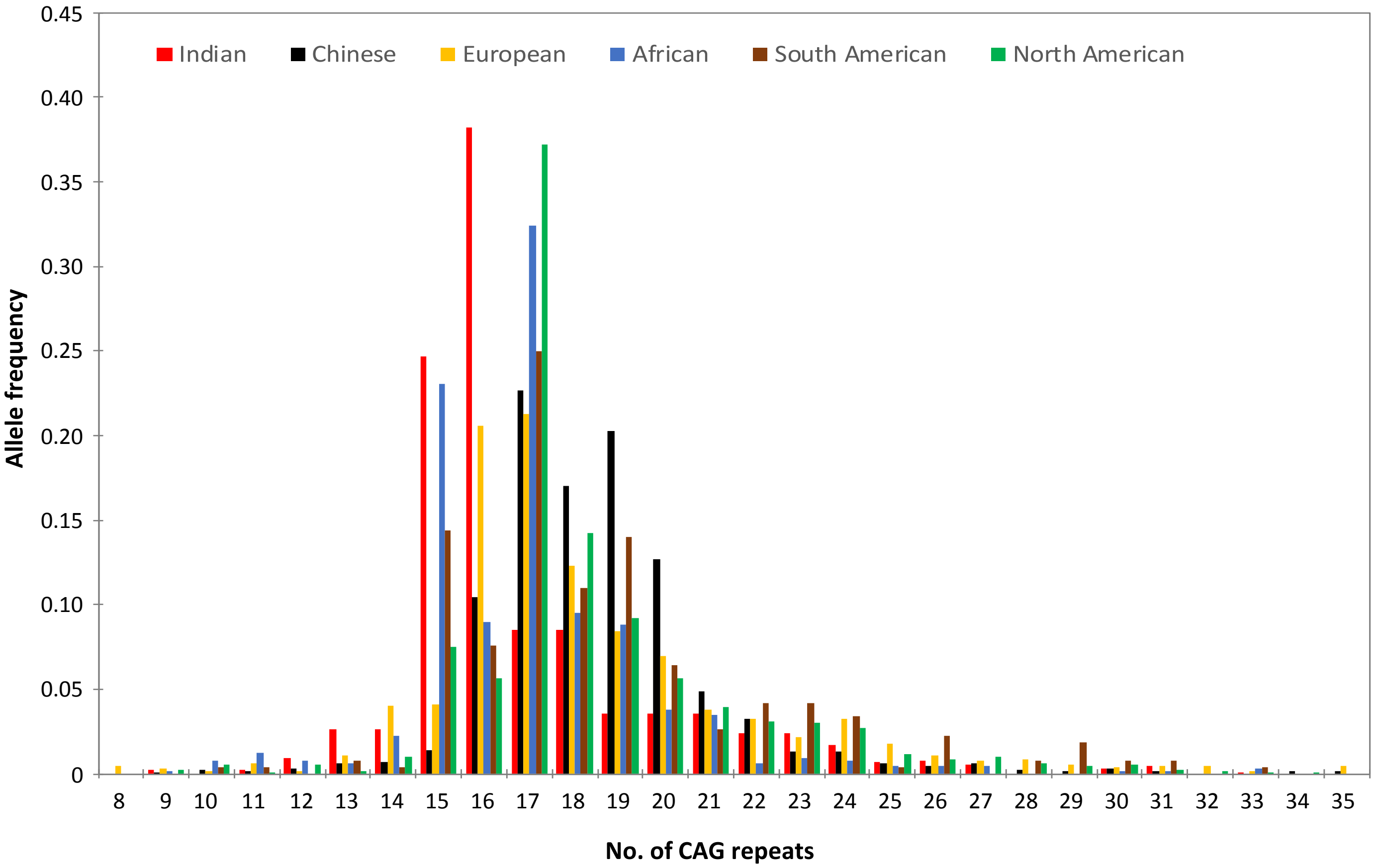

Supplemental Figure S1. *HTT* CAG allele frequency in 6 populations: African, South American, North American, European, Chinese and Indian (references are listed). Although alleles of 33-35 CAGs may be classified as screen-positive when using pHTT(CAG)33 to establish the threshold temperature, such alleles represent only ~0.28% of total alleles in the population, and screen-positive samples require a second-tier CE-based sizing analysis in order to be confirmed as being HD-affected.

References

1. Jiang H, Sun YM, Hao Y, Yan YP, Chen K, Xin SH, et al. Huntingtin gene CAG repeat numbers in Chinese patients with Huntington's disease and controls. Eur J Neurol 2014;21:637-42.
2. Raskin S, Allan N, Teive HA, Cardoso F, Haddad MS, Levi G, et al. Huntington disease: DNA analysis in Brazilian population. Arq Neuropsiquiatr 2000;58:977-85.
3. Semaka A, Kay C, Doty CN, Collins JA, Tam N, Hayden MR. High frequency of intermediate alleles on huntington disease-associated haplotypes in British Columbia's general population. Am J Med Genet B Neuropsychiatr Genet 2012;162:864-71.
4. Saleem Q, Roy S, Murgood U, Saxena R, Verma IC, Anand A, et al. Molecular analysis of Huntington's disease and linked polymorphisms in the Indian population. Acta Neurol Scand 2003;108:281-6.
5. Alonso ME, Yescas P, Cisneros B, Martinez C, Silva G, Ochoa A, Montanez C. Analysis of the (CAG)n repeat causing Huntington's disease in a Mexican population. Clin Genet 1997;51:225-30.
6. Chan V, Yu YL, Chan TP, Yip B, Chang CM, Wong MT, et al. DNA analysis of Huntington's disease in southern Chinese. J Med Genet 1995;32:120-4.
7. Paradisi I, Hernandez A, Arias S. Huntington disease mutation in Venezuela: age of onset, haplotype analyses and geographic aggregation. J Hum Genet 2008;53:127-35.
8. Costa MC, Magalhaes P, Ferreirinha F, Guimaraes L, Januario C, Gaspar I, et al. Molecular diagnosis of Huntington disease in Portugal: implications for genetic counselling and clinical practice. Eur J Hum Genet 2003;11:872-8.
9. Jakab K, Gardian G, Endreffy E, Kalmar T, Bachrati C, Vecsei L, Rasko I. Analysis of CAG repeat expansion in Huntington's disease gene (IT 15) in a Hungarian population. Eur Neurol 1999;41:107-10.
10. Barron LH, Warner JP, Porteous M, Holloway S, Simpson S, Davidson R, Brock DJ. A study of the Huntington's disease associated trinucleotide repeat in the Scottish population. J Med Genet 1993;30:1003-7.
11. Wang CK, Wu YR, Hwu WL, Chen CM, Ro LS, Chen ST, et al. DNA haplotype analysis of CAG repeat in Taiwanese Huntington's disease patients. Eur Neurol 2004;52:96-100.
12. Pramanik S, Basu P, Gangopadhaya PK, Sinha KK, Jha DK, Sinha S, et al. Analysis of CAG and CCG repeats in Huntingtin gene among HD patients and normal populations of India. Eur J Hum Genet 2000;8:678-82.
13. Novelletto A, Persichetti F, Sabbadini G, Mandich P, Bellone E, Ajmar F, et al. Analysis of the trinucleotide repeat expansion in Italian families affected with Huntington disease. Hum Mol Genet 1994;3:93-8.
14. Baine FK, Kay C, Ketelaar ME, Collins JA, Semaka A, Doty CN, et al. Huntington disease in the South African population occurs on diverse and ethnically distinct genetic haplotypes. Eur J Hum Genet 2013;21:1120-7.
